# Human BAT Possesses Molecular Signatures That Resemble Beige/Brite Cells

**DOI:** 10.1371/journal.pone.0049452

**Published:** 2012-11-16

**Authors:** Louis Z. Sharp, Kosaku Shinoda, Haruya Ohno, David W. Scheel, Emi Tomoda, Lauren Ruiz, Houchun Hu, Larry Wang, Zdena Pavlova, Vicente Gilsanz, Shingo Kajimura

**Affiliations:** 1 UCSF Diabetes Center and Department of Cell and Tissue Biology, University of California San Francisco, San Francisco, California, United States of America; 2 Department of Radiology, Childrens Hospital Los Angeles, University of Southern California, Los Angeles, California, United States of America; 3 Department of Pathology, Childrens Hospital Los Angeles, University of Southern California, Los Angeles, California, United States of America; Graduate School of Medicine, the University of Tokyo, Japan

## Abstract

Brown adipose tissue (BAT) dissipates chemical energy and generates heat to protect animals from cold and obesity. Rodents possess two types of UCP-1 positive brown adipocytes arising from distinct developmental lineages: “classical” brown adipocytes develop during the prenatal stage whereas “beige” or “brite” cells that reside in white adipose tissue (WAT) develop during the postnatal stage in response to chronic cold or PPARγ agonists. Beige cells’ inducible characteristics make them a promising therapeutic target for obesity treatment, however, the relevance of this cell type in humans remains unknown. In the present study, we determined the gene signatures that were unique to classical brown adipocytes and to beige cells induced by a specific PPARγ agonist rosiglitazone in mice. Subsequently we applied the transcriptional data to humans and examined the molecular signatures of human BAT isolated from multiple adipose depots. To our surprise, nearly all the human BAT abundantly expressed beige cell-selective genes, but the expression of classical brown fat-selective genes were nearly undetectable. Interestingly, expression of known brown fat-selective genes such as *PRDM16* was strongly correlated with that of the newly identified beige cell-selective genes, but not with that of classical brown fat-selective genes. Furthermore, histological analyses showed that a new beige cell marker, CITED1, was selectively expressed in the UCP1-positive beige cells as well as in human BAT. These data indicate that human BAT may be primary composed of beige/brite cells.

## Introduction

Brown adipose tissue (BAT) is specialized to generate heat by dissipating chemical energy as a defense against cold and obesity. Thermogenesis in the BAT is mediated through a brown fat-specific mitochondrial protein, uncoupling protein 1 (UCP1), which plays an important role in the control of energy homeostasis. Indeed, loss of UCP1 causes cold-intolerance as well as obesity at thermoneutality in mice [1], [2]. Importantly, recent studies using non-invasive imaging technologies such as ^18^fluoro-labeled 2-deoxy-glucose positron emission tomography (^18^FDG-PET) scanning and MRI scanning clearly demonstrated that adult humans have significant amounts of active brown fat deposits [3], [4], [5], [6], [7], [8], [9]. A histological study also identified brown fat precursors in the BAT from adult humans [10]. Furthermore, the glucose uptake capacity in the BAT correlates inversely with adiposity, indicating that variation in the amount and/or thermogenic activity of BAT may contribute to the propensity for weight gain in humans [11], [12].

Current evidence suggests that there are at least two types of brown adipocytes that arise from distinct developmental lineages in rodents. “Classical” brown adipocytes that reside in the interscapular BAT depot and perirenal regions develop during the prenatal stage from *Myf-5* positive myoblast precursors [13]. Global gene expression analyses indicate that the brown fat precursors in the interscapular BAT show a gene signature that resembles that of skeletal muscle cells [14]. In addition, a proteome study has shown that mitochondria from the interscapular BAT are most similar to their counterparts in skeletal muscle at transcriptional and protein levels [15]. These *Myf-5* derived precursors differentiate into brown adipocytes through the action of two transcriptional factors, PRDM16 and C/EBP-β [13], [16], although it remains unknown whether the *Myf-5* positive cells clonally give rise to brown adipocytes and myocytes.

Another type of brown adipocytes is sporadically found as a copious cluster in the white adipose of adult animals that have been chronically exposed to cold or to PPARγ ligands (so called beige or brite cells). These “inducible” brown adipocytes possess the morphological and biochemical characteristics of classical brown adipocytes, including the expression of UCP1 and multilocular lipid droplet [17], however, they arise from a non-*Myf-5* lineage [13]. A recent study has shown that PDGFRa-positive bi-potent progenitors in the abdominal WAT give rise to brown adipocytes in response to beta-adrenergic stimulation *in vivo* [18]. Cannon and Nedergaard’s group has also shown that these brite cells express several well-known brown fat-selective genes such as *Ucp1*, but do not express myocyte-enriched genes, indicating that these two types of brown adipocytes have distinct expression profiles [19], [20]. Of note, Kozak’s group has implied that signaling pathways to control these beige cells in the WAT are different from classical brown adipocytes in the interscapular BAT depots [21], [22], [23]. Importantly, increases in number of beige cells in WAT are associated well with a protection against diet induced obesity and metabolic diseases [24], [25], [26]. Hence, an emerging interest in the field of brown fat biology is to characterize the molecular and functional signatures of the beige cells *in vivo*. In particular, the relevance of the beige cells in humans must be now addressed.

In this study, we asked if humans possess beige cells. To this end, we first employed microarray analysis to define the unique gene sets that are distinctively expressed in the *Myf-5* derived classical brown adipocytes and in the non-*Myf-5* beige/brite cells in mice. Subsequently we applied the transcriptional data to human adipose tissues and determined the molecular signatures of human BAT. Surprisingly, nearly all the human BAT isolated from multiple locations abundantly expressed genes unique to beige cells, correlating very well with several known brown fat-selective genes such as *PRDM16* and *PGC1α*. On the contrary, the classical brown adipocyte-selective genes were nearly undetected in human BAT. Our transcriptional and histological analyses indicate that human BAT possesses a molecular signature of beige cells.

## Materials and Methods

### Animals

All animal experiments were performed according to procedures approved by UCSF’s Institutional Animal Care and Use Committee for animal care and handling. C57BL/6 mice (Jackson Laboratories) were used for the animal experiments. To induce the browning of white fat in mice, CL316243 at a dose of 1 mg/kg or saline control was injected intraperitoneally for 8 days into 6–8 week-old mice. Inguinal adipose tissue was isolated, fixed in 4% paraformaldehyde, and embedded in paraffin for histological analysis.

### Immunohistochemistry

Paraffin-embedded sections were cut to 7 µm sections. They were subjected to citrate-based antigen retrieval using Antigen Retrieval Citra Solution (Biogenex) and incubated for 1 hour at room temperature with rabbit polyclonal antibodies against CITED1 at a dilution of 1∶500 (Abcam) or UCP1 at a dilution of 1∶1000 (Abcam) or IgG as negative control. Secondary detection was performed for 30 minutes at room temperature using anti-rabbit Alexa 488 and Alexa 594 (Invitrogen). Images were captured using a Zeiss Axioskop 2 Widefield microscope.

### Human BAT Samples and MRI Imaging

The protocol for the human study was approved by the Institutional Review Board of Children’s Hospital Los Angeles (CHLA), where the imaging and postmortem examinations were performed. Written informed consent was obtained from all parent/legal guardian(s) in the presence of a witness. MRI scanning was performed in the patients to identify the BAT as previously described [9], [27]. Briefly, 13 post-mortem examinations of children ranging from 3-day to 18-years-old were scanned using MRI implemented on a 3 T whole-body human platform (Achieva, R2.6.3, Philips Healthcare, Cleveland, OH). The mDIXON (Philips Healthcare, Cleveland, Ohio) chemical-shift water-fat multi-echo pulse sequence provided by the manufacturer was utilized in this work using an 8- or 16-channel coil. A consistent acquisition voxel size of 1 mm × 1 mm × 2 mm was maintained across all subjects. For the purpose of this study, the fat fraction (FF) of adipose tissue in the chest and the abdomen was calculated using multi-echo chemical-shift water-fat MRI algorithms as previously described [9], [27]. Image analysis and quantification of FF maps were performed with SliceOmatic (Tomovision, Inc., Magog, Canada) segmentation software. Based on the MRI FF, biopsies of adipose tissue were obtained within 24 hours after death by a pediatric pathologist at sites highly suggestive of BAT. Routine histological and immunohistochemical assays and molecular analysis of tissue samples were performed at the Department of Pathology at CHLA and at UCSF.

### Cell Culture

Primary stromal vascular (SV) cells were isolated from inguinal WAT or from interscapular BAT of the same C57BL/6 mice using Collagenase D (1.5 u/ml) and Dispase II (2.4 u/ml). To clearly define the molecular signatures of PPARγ ligand-induced beige cells, we used C57BL/6 mice that possess less beige cells in the absence of environmental stimuli. SV cells were plated in collagen coated culture dishes in DMEM/F12 medium (D-glucose 17.51 mM). The confluent cells were subsequently differentiated in DMEM/F12 medium containing 10% FBS, 0.5 mM isobutylmethylxanthine, 125 nM indomethacin, 1 mM dexamethasone, 850 nM insulin and 1 nM T3. To induce beige cell differentiation, a specific PPARγ agonist rosiglitazone at 1 µM were added in the medium [28]. Two days after induction, cells were switched to the maintenance medium containing 10% FBS, 850 nM insulin and 1 nM T3 for additional 4–5 days until cells were fully differentiated to mature adipocytes. R2F primary skin fibroblasts isolated from human newborn foreskin were cultured following the methods described elsewhere [29].

### RNA Isolation and Gene Expression Analysis

Total RNA was isolated from tissues using the Trizol reagent (Invitrogen) or RiboZol reagent (AMRESCO) following the manufacturer’s protocol. Quality of RNA from all the samples was checked by spectrophotometer. Samples that did not clear the quality control (O.D.260/280<1.8) were excluded from the analyses. Reverse transcription reactions were performed using IScript cDNA synthesis kit (Bio-Rad). The sequences of primers used in this study can be found in Table S1. Quantitative real-time PCR (qRT-PCR) was performed with SYBR green fluorescent dye using an ABI ViiA™7 PCR machine. Relative mRNA expression was determined by the ΔΔ-Ct method using TATA-binding protein (TBP) as an endogenous control to normalize samples. For a microarray analysis, Affymetrix GeneChip Mouse Genome 430 2.0 array was used according to established methods [30]. The array data were analyzed using the DNA-Chip Analyzer (dChip) software. The statistical significance of differences in gene expression was assessed by unpaired t-test (*P*<0.05). Microarray data has been deposited in Gene Expression Omnibus (GEO): GSE35011. Patient and sample information for the tissues used in the expression analyses were provided in Table S2.

### Heatmap Visualization

The measured mRNA expression were clustered according to the gene group and visualized as a heatmap representation using Multi Experiment Viewer [31]. Expression levels of all genes were visualized in the same green-black-red scheme in order to evaluate relative expression levels in all samples.

### Statistical Analyses

Hierarchical clustering was performed with average linkage method using Pearson correlation as distance metric. Integration of correlation coefficient among gene group was done using average of weighted Fisher’s z-score. We transformed each coefficient into z-score and computed average and then inverse transformed. Test of population correlation coefficient was conducted by Z-test. To detect outliers, we used box-and-whisker plot. Samples beyond the whiskers of the plot were considered outlier. All these statistical analysis were performed using the JMP software version 9 (SAS Institute) or R version 2.11.1.

## Results

### Distinct Molecular Signatures between *Myf-5* Derived Brown Adipocytes and Non-*Myf-5* Derived Beige Cells

Classical brown adipocytes in interscapular BAT (*Myf-5* derived) and “inducible” beige cells in WAT (non-*Myf-5* derived) have distinct developmental origins, although both cell types have morphological and biochemical characteristics of brown fat such as the expression of UCP1 [13]. This raises an important question as to how similar the two types of brown adipocytes are at molecular and functional levels. To this end, we first employed microarray analysis to systematically determine the transcriptional signatures unique to each cell type. To avoid contamination from non-adipocytes, the stromal vascular (SV) fraction was isolated from interscapular BAT or inguinal WAT and fully differentiated *in vitro*. A subset of the SV cells from inguinal WAT were differentiated in the presence of rosiglitazone at 1 µM to induce the development of beige cells [28]. At least 95% of the cells differentiated into mature adipocytes under this culture condition.

**Figure 1:**
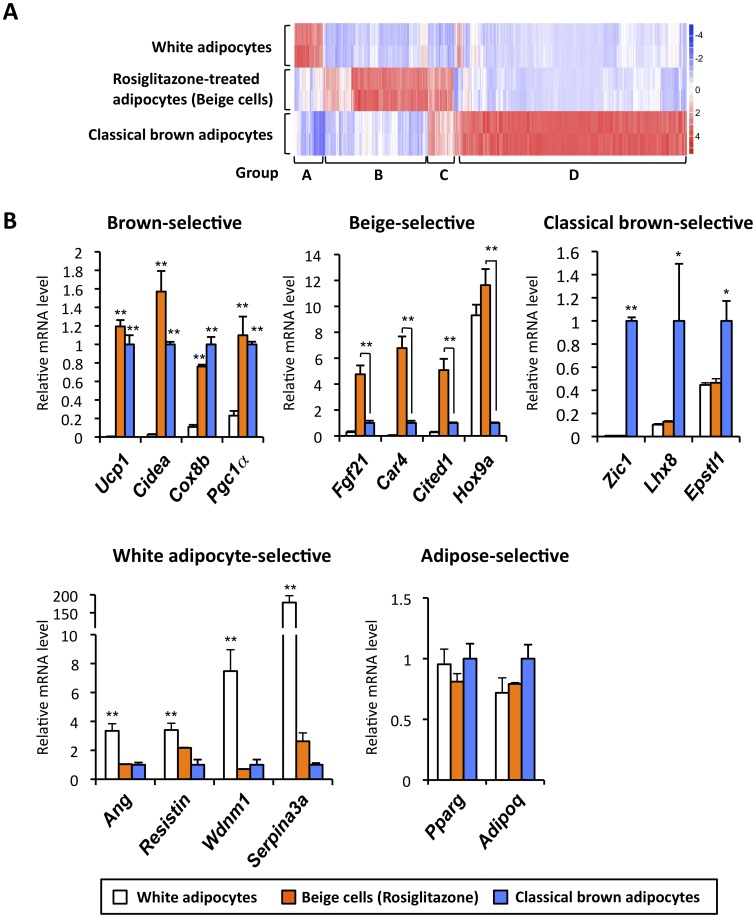
Distinct molecular signatures in *Myf-5* derived classical brown adipocytes and non-*Myf-5* derived beige cells. (**A**) Microarray analysis was performed in fully differentiated brown adipocytes from interscapular BAT (classical brown adipocytes), white adipocytes isolated from inguinal WAT and rosiglitazone-treated adipocytes (beige cells). The color scale shows the fold changes in mRNA expression levels of the genes in blue (down-regulation)-white-red (up-regulation) scheme. (**B**) mRNA levels for the indicated genes were analyzed by qRT-PCR. Relative expression levels in the brown adipocyte were set as 1.0. * *P*<0.05, **P<0.01. Data are expressed as means ± SD.

**Figure 2:**
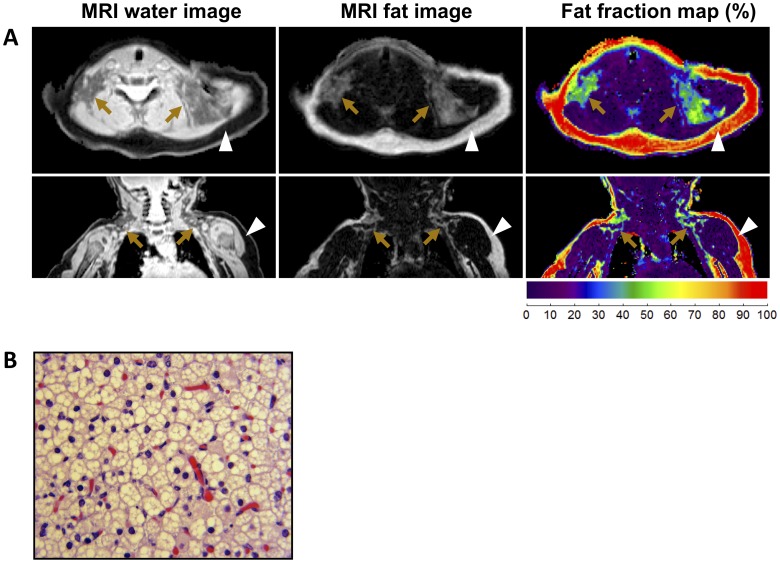
Isolation of human BAT from multiple BAT depots. (**A**) Axial (top) and coronal (bottom) MRI scanning images illustrating bilateral supraclavicular BAT depots from chemical-shift water-fat MRI in a 4-months-old female. BAT depots are denoted by arrows and subcutaneous WAT depots are denoted by arrowheads. The first column shows the water images. The second column shows the fat images. The *third* column shows the fat fraction map of water content, which translates to a lower fat fraction in BAT than WAT. The coronal images were obtained anteriorly at the level of the humeral epiphysis. (**B**) The isolated BAT depots were fixed and stained by H&E. The sample was obtained from the left supraclavicular area of the same 4-month old female shown in the MR images in Fig. 2A. Note that nearly all the isolated BAT samples consisted of brown adipocytes containing multilocular lipid droplets.

Subsequently, mRNA expression of genes was compared between classical brown adipocytes, inguinal white adipocytes and rosiglitazone-treated adipocytes (beige cells). As shown in Fig.1A, we identified a total of 496 genes (>2 fold, *P*<0.05) that were differentially expressed among the three groups. These genes could be clustered into four groups as follows: Group A included 32 genes whose expression was enriched in inguinal white adipocytes; Group B contained 141 genes whose expression was enriched in rosiglitazone-treated adipocytes (beige cells); Group C included 29 genes enriched both in classical brown adipocytes and in beige cells; Group D contained 294 genes enriched in classical brown adipocytes. For instance, as verified by qRT-PCR, well-known brown fat-selective genes, such as *Ucp1*, *Cidea*, *Cox8b* and *Pgc1α* were expressed at similar levels both in classical brown adipocytes and in rosiglitazone-treated adipocytes (Fig.1B, upper left). Such “common” brown fat-selective genes for the classical brown adipocytes and rosiglitazone-treated adipocytes (beige cells) represent only a small portion of the total (9%, 29/323 genes in classical brown adipocytes or 17%, 29/170 genes in beige cells). In contrast, group B were genes whose expression was enriched only in the rosiglitazone-induced beige cells (listed in Table S3). This includes fibroblast growth factor 21 (*Fgf21*), carbonic anhydrase 4 (*Car4*), and Cbp/p300-interacting transactivator with Glu/Asp-rich carboxy-terminal domain 1 (*Cited1*). Expression of homeo box gene 9 (*Hox9a*) was enriched in both white adipocytes and beige cells (Fig.1B, upper middle). Group D includes genes that are highly enriched only in classical brown adipocytes (listed in Table S4). This group contained zinc finger protein of the cerebellum 1 (*Zic1*), LIM homeobox protein 8 (*Lhx8*), and epithelial stromal interaction 1 (*Epstl1*) (Fig.2B, upper right). This is consistent with the previous studies by Cannon and Nedergaard’s group [19], [20]. Expression of angiotensinogen (*Ang*), *resistin*, WDNM1-like protein (*Wdnm1*), and *Serpina3a*, as found in Group A and reported in previous studies [32], [33], was enriched in white adipocytes (Fig.1B, lower left). On the other hand, expression of general adipogenic genes shared in differentiated white adipocytes, beige cells and brown adipocytes, such as *Pparg* and Adiponectin (*Adipoq*) were expressed at similar levels, suggesting that adipocyte differentiation was similar in three groups. These data clearly suggest that the two types of brown adipocytes have distinct characteristics at the molecular levels.

### Isolation of Human BAT from Multiple Depots

Recent advance in MRI scanning technology allowed us to identify the BAT in multiple adipose depots in humans [9]. To isolate the BAT from multiple locations in given individuals, we first scanned 13 patients by MRI. Fig2A illustrates axial (top panels) and coronal (bottom panels) images of MRI results in a 4-months-old female. As shown in the first column (water image), BAT is hyperintense, or brighter image than WAT, since BAT contains greater intracellular water content than WAT. On the contrary, in the second column (fat image), BAT is hypointense or darker, than WAT, due to its lower intracellular fat content. Collectively, as shown in the third column, the difference in fat and water content translates to a lower fat fraction in BAT than WAT with a fraction of about 30–60%. Of note, nearly all of the subcutaneous compartments were red and had a fat fraction between 80–100%, which was indicative of white adipose tissue. In contrast, the adipose tissues in the supraclavicular and intra-abdominal areas showed fat fraction characteristics of brown fat. The BAT depots were located in the subcutaneous supraclavicular areas, posterior mediastinum, retroperitoneal, intraabdominal and mesenteric depots, with thigh tissue samples for 4 of the 13 patients. In contrast to rodents in which BAT is preferentially located in the interscapular area, this tissue in humans is primarily recognized in the cervical-supraclavicular area, where it is present across a wide range of ages [3], [4], [34], [35]. On occasion, newborns and very young infants present BAT in the interscapular area, but this depot is replaced by white fat shortly after.

Subsequently, the identified BAT depots were biopsied for histological analysis. As represented in Fig. 2B and also previously reported (Gilsanz et al. 2012), H&E staining analyses showed that most of these tissues in supraclavicular areas, posterior mediastinum, retroperitoneal, intraabdominal and mesenteric depots consisted of *bona-fide* brown adipocytes containing multilocular lipid droplets.

### Human BAT Expresses a Large Array of Beige Cell-selective Genes

To examine the molecular signatures of human BAT, total RNA was isolated from the human BAT samples isolated from multiple locations, including subcutaneous supraclavicular areas, posterior mediastinum, retroperitoneal, intraabdominal, mesenteric depots and thigh tissues. As controls, total RNA was also isolated from white adipose tissue and from smooth muscle and included in the analysis. Subsequently, we measured the expression of classical brown adipocyte-selective or beige cell-selective genes that were identified in Fig.1. A recent study by Spiegelman’s group has reported that three genes, *CD137, TMEM26*, and *TBX1* were enriched in the beige cells but not in the classical brown adipocytes nor in the white adipocytes [36]. These genes were therefore included in the expression analyses.

**Figure 3:**
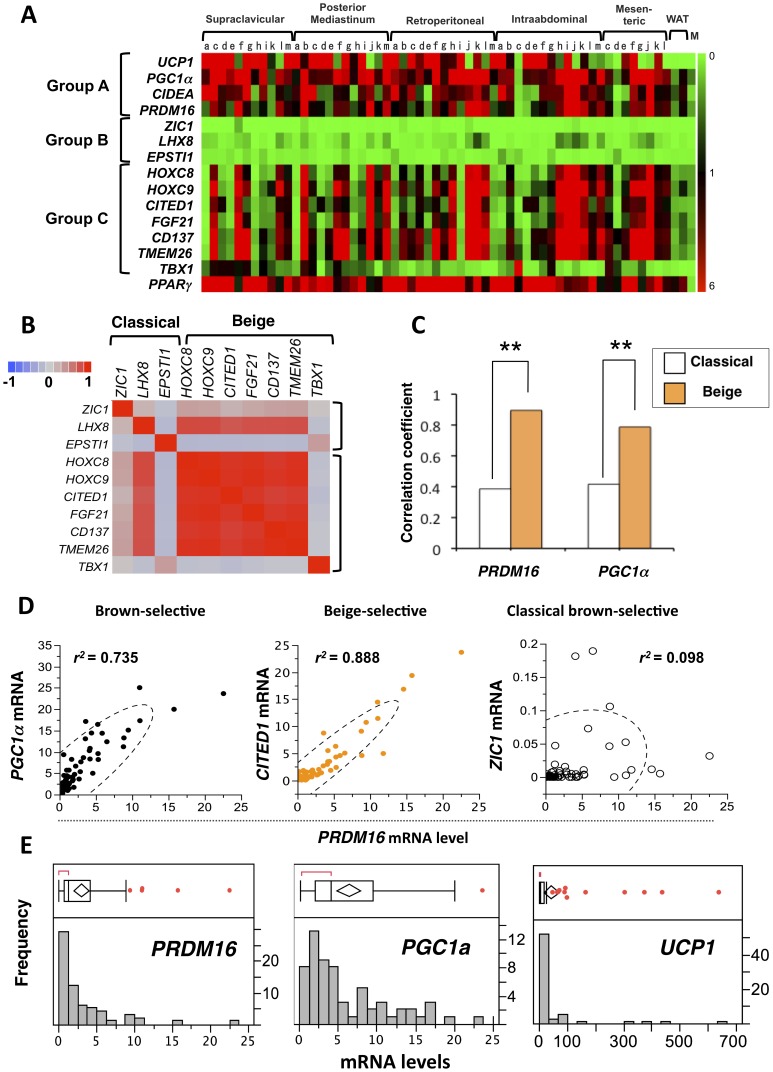
Transcriptional analyses of human BAT. (**A**) Expression profiles of the common brown fat-selective genes (Group A), classical brown fat-selective genes (Group B), and beige cell-selective genes (Group C) in human BAT from multiple adipose depots. The color scale shows the mRNA levels of the genes in green (low or no-expression)-black-red (high-expression) scheme. All genes are shown in the identical scale. WAT: white adipose tissue. M: smooth muscle. Each alphabet indicates the tissues from the same patient. (**B**) Correlation matrix of beige-selective and classical brown-selective gene expression. The color scale shows the Pearson correlation between each gene mRNA expression in blue (negative correlation, −1.0)-gray (no correlation, 0)-red (positive correlation, 1.0) scheme. (**C**) Strength of correlation with representative group A gene (*PRDM16* and *PGC1a*) and classical-brown-selective genes or beige cell-selective genes. **P<0.01 relative to representative group B gene. (**D**) Correlation between the mRNA expression of *PRDM16* and *PGCa1* (left), *CITED1* (middle), and *ZIC1* (right). These genes represent each gene class. Dotted lines denote density ellipse (95% confidence interval of plot). (**E**) Box-and-whisker plot (upper) and histogram (bottom) to graphically summarize the distribution of each gene expression levels. The lines extending from end of the box (quartile) are whiskers, which edge is the outermost data point(s) that fall within the distance defined by quartile ±1.5*(IQR). Values beyond the whiskers, denoted by a red point indicate outlier samples.

Fig.3A shows the expression profiles of the common brown fat-selective genes (Group A), classical brown fat-selective genes (Group B), and beige cell-selective genes (Group C). Expression of *PPARγ*, a master regulator of adipocyte development, was also measured to validate that these samples were indeed adipose. To our surprise, expression of classical brown fat markers was nearly undetectable, whereas beige cell markers were abundantly expressed in early all human BAT. Notably, beige-selective transcription factors (*HoxC8, HoxC9* and *CITED1*) were highly expressed in human BAT. The trend of expression profile was consistent after normalizing the each value with that in non-adipocyte cells such as primary skin fibroblasts (Fig. S1).

Next we systematically asked, based on the gene expression profile, how similar human BAT was to the classical brown adipocytes or to the beige cells. First, we used Pearson correlation to evaluate the similarities of expression pattern among classical brown- and beige-selective genes. As shown in Fig. 3B, all the beige selective genes except *TBX1* showed high (*r*>0.87) cross-correlations, justifying the analysis based on a set of gene group designation. We then integrated correlation coefficient between established BAT markers such as *PRDM16* and each classical brown- or beige-selective gene into a “gene-set correlation coefficient” according to the groupings; this allowed us to analyze the expression data as “gene-sets” rather than single-gene-based analysis that is sensitive to outlier samples inherent in human postmortem samples. As shown in Fig. 3C (left), the correlation coefficient between *PRDM16* and beige cell-selective genes was significantly higher, as compared with those between *PRDM16* and classical brown-selective genes (*P*<0.01, Z-test). Similarly, this difference was highly significant in *PGC1a*, a well-known common brown adipocyte-enriched gene (*P*<0.01, Fig. 3C, right). Furthermore, the expression profiles of Group B and C was clearly different at each individual gene level; As shown in Fig.3D, mRNA expression of *PRDM16* correlated very well with that of *PGC1a* (left), and also with that of beige cell-selective genes including *CITED1* (middle), but not with that of classical brown fat genes such as *ZIC1* (right). mRNA expression of *UCP1* was not included in this analysis because outlier plot analysis detected too many outlier samples in *UCP1* mRNA levels, partly due to insufficient number of samples. Therefore this did not allow us to run an appropriate correlation analysis using *UCP1* mRNA levels. On the other hand, most samples fell within the range of the whisker in *PRDM16* and *PGC1a* mRNA levels. (Fig. 3E). Taken together, these data indicate that molecular signature of human BAT resembles beige cells.

### CITED1 is a New Marker for Beige Cells in Mice and in Humans

**Figure 4:**
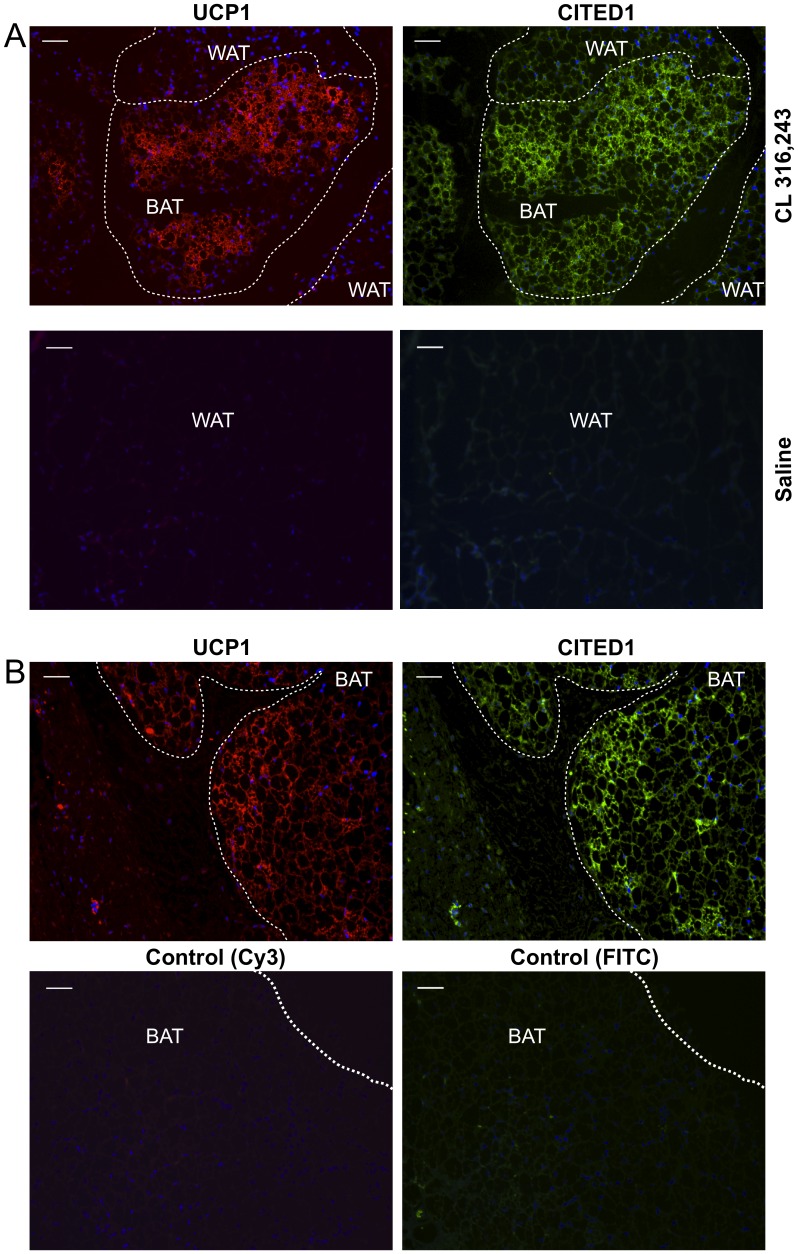
Expression of a new beige cell marker CITED1 in mice and in humans. (**A**) Immunohistochemistry of UCP1 (left) and CITED1 (right) in serial sections of WAT in mice that were treated with CL316243 at a dose of 1 mg/kg (upper panels) or saline control (lower panels) for 8 days**.** Scale bar, 50 µm. (**B**) Immunohistochemistry of UCP1 (left) and CITED1 (right) in serial sections of human BAT. Lower panels show IgG negative controls of immunohistochemistry for UCP1 (Cy3) and for CITED1 (FITC), respectively in human BAT. Scale bar, 50 µm.

Although the H&E staining showed that the human BAT samples mostly consisted of brown adipocytes containing multilocular lipid droplets (Fig.2B), qRT-PCR based expression analyses could not exclude the possibility that expression of the beige-selective genes might come from non-brown adipocytes in a given tissue. To address this, we performed immunohistochemistry of a new beige marker, CITED1 in mice and in humans. UCP1 immunohistochemistry was also performed in serial sections to identify the brown adipocytes. CITED1 was originally identified in a murine melanoma cell line [37] and in human thyroid carcinoma [38], [39]. It has been reported that CITED1 acts as a transcriptional co-activator for estrogen receptor [40]. As shown in Fig.4A, CITED1 was selectively expressed in the UCP1-positive beige cells in the inguinal WAT in mice chronically treated with a β3 agonist CL316243 (upper panels). In contrast, there were almost no UCP1-positive or CITED1-positive cells in the inguinal WAT in mice treated with saline control (Fig.4A lower panels). Consistent with this, CITED1 was well expressed in the UCP1-positive brown adipocytes in human BAT (Fig. 4B, upper panels). Negative controls for the immunofluorescent stainings of UCP1 and CITED1 (Cy3 and FITC, respectively) confirmed the specificity of the staining (Fig. 4B, lower panels). These data indicate that CITED1 serves as a marker for beige cells both in mice as well as in humans.

## Discussion

Two types of brown adipocytes with distinct developmental origins has been previously described in mice: *Myf5*-derived pre-exiting brown adipocytes in the interscapular BAT and non-*Myf5*-derived beige cells that emerge in the subcutaneous WAT in response to chronic cold exposure or to PPARγ agonists. The present study characterized the unique molecular signatures for the two types of brown adipocytes, which allowed us to ask whether humans possess beige cells. To our surprise, a number of beige cell-selective genes were abundantly expressed in most, if not all, human BAT isolated from multiple adipose depots, correlating very well with the expression of known common brown fat-selective genes such as *PRDM16* and *PGC1α*, but not with that of classical brown fat-selective genes. Although the current study is limited to molecular analyses, the present data indicate that human BAT may resemble beige cells. This view is in consistent with the recent report by Spiegelman’s group showing that three beige cell markers CD137, TMEM26, and TBX1 are enriched in the human UCP1-positive brown adipocytes [36]. To be noted, histological analyses of human BAT showed that UCP1-positive brown adipocytes formed a copious cluster and resided together with white adipocytes. These clusters are a morphological characteristic of beige cells in the mouse WAT [3], [4], [5], [6], [41]. Global analyses of human brown adipocytes will further clarify its cellular identity and developmental origin in detail. The number of patient examined in this study was not sufficient to critically examine the age-related changes in the expression of brown fat-selective genes, however, we will investigate this point in our future studies.

We found strong correlations among beige cell-selective gene expressions in human BAT, indicating that there is a dominant regulatory pathway for beige cell development. To be noted, expressions of *PRDM16* correlated very well with those of most beige cell genes. PRDM16 is a 140 kDa zinc-finger protein containing PR-(PRD1-BF-1-RIZ1 homologous) domain that was originally identified at a chromosomal breakpoint in human myeloid leukemia cells [42], [43]. Indeed, it has been shown that PRDM16 expression in primary and immortalized white adipocytes is sufficient to activate a large array of brown-fat selective genes *via* interacting with several transcriptional factors such as PPARγ and PGC1α [26], [32], [44]. In addition, PRDM16 expression together with C/EBPβ is able to convert human foreskin-derived fibroblasts into UCP1-positive brown adipocytes [16]. Our recent study has also shown that PRDM16 is required for the development of beige cells (so called “browning” of white fat) in response to PPARγ agonists in mice [28]. These studies indicate that PRDM16 is one of the dominant factors for the formation of human BAT, however, the requirement of PRDM16 in humans remains to be tested.

The development of beige cells can occur in adult mice and is highly inducible in response to a variety of environmental cues and endogenous hormones, therefore, this cell type possesses a tremendous potential as a therapeutic target. The present study indicates the relevance of this cell type in humans, further supporting this idea. One of the emerging questions would be to understand the developmental processes and molecular control of the beige cells. Wu et al., have recently cloned beige-cell lines from mouse inguinal WAT and showed that beige-precursors expressed unique sets of genes that could distinguish beige cells from white or classical brown adipocytes [36]. Meanwhile, Cinti’s group has shown that the browning of white fat in response to cold is mainly due to the transdifferentiation of mature white adipocytes into brown adipocytes in mice [45]. It needs to be addressed how proliferation of beige cell precursors and white-to-brown adipocyte transdifferentiation contributes to the development of human BAT.

Previous attempts to activate thermogenesis in existing BAT by beta3-adrenergic receptor agonists have been unsuccessful in humans [46]. Because human BAT possesses molecular characteristics of beige cells, a future therapeutic strategy for human obesity would be to induce the browning of white fat. For example, inhibition of TGF-β signaling by neutralizing antibodies sufficiently increases whole body energy expenditure and counteracts diet-induced obesity, at least in part, by browning white fat in mice [47], [48]. Alternatively, using endogenous hormones such as FGF21 [49], natriuretic peptides [50], prostaglandin [51], [52], or Irisin [53] to promote browning of white fat would be a plausible approach.

## Supporting Information

Figure S1 **Expression profile after normalizing the each gene mRNA level with that of primary skin fibroblasts.**
(TIF)

Table S1 **Primer sequences used in quantitative real-time PCR.**
(PDF)

Table S2 **Patient and sample information for the tissues used in the expression analyses.**
(PDF)

Table S3 **Genes enriched in rosiglitazone-inducible beige/brite cells.**
(PDF)

Table S4 **Genes enriched in classical brown adipocytes**
(PDF)

## References

[1] Enerback S, Jacobsson A, Simpson EM, Guerra C, Yamashita H, et al (1997) Mice lacking mitochondrial uncoupling protein are cold-sensitive but not obese. Nature 387: 90–94. 10.1038/387090a0 9139827

[2] Feldmann HM, Golozoubova V, Cannon B, Nedergaard J (2009) UCP1 ablation induces obesity and abolishes diet-induced thermogenesis in mice exempt from thermal stress by living at thermoneutrality. Cell Metab 9: 203–209. 19187776 10.1016/j.cmet.2008.12.014

[3] Cypess AM, Lehman S, Williams G, Tal I, Rodman D, et al (2009) Identification and importance of brown adipose tissue in adult humans. N Engl J Med 360: 1509–1517. 19357406 10.1056/NEJMoa0810780 PMC2859951

[4] van Marken Lichtenbelt WD, Vanhommerig JW, Smulders NM, Drossaerts JM, Kemerink GJ, et al (2009) Cold-activated brown adipose tissue in healthy men. N Engl J Med 360: 1500–1508. 19357405 10.1056/NEJMoa0808718

[5] Virtanen KA, Lidell ME, Orava J, Heglind M, Westergren R, et al (2009) Functional brown adipose tissue in healthy adults. N Engl J Med 360: 1518–1525. 19357407 10.1056/NEJMoa0808949

[6] Saito M, Okamatsu-Ogura Y, Matsushita M, Watanabe K, Yoneshiro T, et al (2009) High incidence of metabolically active brown adipose tissue in healthy adult humans: effects of cold exposure and adiposity. Diabetes 58: 1526–1531. 19401428 10.2337/db09-0530 PMC2699872

[7] Nedergaard J, Bengtsson T, Cannon B (2007) Unexpected evidence for active brown adipose tissue in adult humans. Am J Physiol Endocrinol Metab 293: E444–452. 17473055 10.1152/ajpendo.00691.2006

[8] Yoneshiro T, Aita S, Matsushita M, Kameya T, Nakada K, et al (2011) Brown adipose tissue, whole-body energy expenditure, and thermogenesis in healthy adult men. Obesity (Silver Spring) 19: 13–16. 20448535 10.1038/oby.2010.105

[9] Hu HH, Tovar JP, Pavlova Z, Smith ML, Gilsanz V (2012) Unequivocal identification of brown adipose tissue in a human infant. J Magn Reson Imaging 35: 938–942. 22180228 10.1002/jmri.23531 PMC3310283

[10] Zingaretti MC, Crosta F, Vitali A, Guerrieri M, Frontini A, et al (2009) The presence of UCP1 demonstrates that metabolically active adipose tissue in the neck of adult humans truly represents brown adipose tissue. Faseb J 23: 3113–3120. 19417078 10.1096/fj.09-133546

[11] Enerback S (2010) Human brown adipose tissue. Cell Metab 11: 248–252. 20374955 10.1016/j.cmet.2010.03.008

[12] Nedergaard J, Cannon B (2010) The changed metabolic world with human brown adipose tissue: therapeutic visions. Cell Metab 11: 268–272. 20374959 10.1016/j.cmet.2010.03.007

[13] Seale P, Bjork B, Yang W, Kajimura S, Chin S, et al (2008) PRDM16 controls a brown fat/skeletal muscle switch. Nature 454: 961–967. 18719582 10.1038/nature07182 PMC2583329

[14] Timmons JA, Wennmalm K, Larsson O, Walden TB, Lassmann T, et al (2007) Myogenic gene expression signature establishes that brown and white adipocytes originate from distinct cell lineages. Proc Natl Acad Sci U S A 104: 4401–4406. 17360536 10.1073/pnas.0610615104 PMC1810328

[15] Forner F, Kumar C, Luber CA, Fromme T, Klingenspor M, et al (2009) Proteome differences between brown and white fat mitochondria reveal specialized metabolic functions. Cell Metab 10: 324–335. 19808025 10.1016/j.cmet.2009.08.014

[16] Kajimura S, Seale P, Kubota K, Lunsford E, Frangioni JV, et al (2009) Initiation of myoblast to brown fat switch by a PRDM16-C/EBP-beta transcriptional complex. Nature 460: 1154–1158. 19641492 10.1038/nature08262 PMC2754867

[17] Frontini A, Cinti S (2010) Distribution and development of brown adipocytes in the murine and human adipose organ. Cell Metab 11: 253–256. 20374956 10.1016/j.cmet.2010.03.004

[18] Lee YH, Petkova AP, Mottillo EP, Granneman JG (2012) In vivo identification of bipotential adipocyte progenitors recruited by beta3-adrenoceptor activation and high-fat feeding. Cell Metab 15: 480–491. 22482730 10.1016/j.cmet.2012.03.009 PMC3322390

[19] Walden TB, Hansen IR, Timmons JA, Cannon B, Nedergaard J (2012) Recruited vs. nonrecruited molecular signatures of brown, “brite,” and white adipose tissues. Am J Physiol Endocrinol Metab 302: E19–31. 21828341 10.1152/ajpendo.00249.2011

[20] Petrovic N, Walden TB, Shabalina IG, Timmons JA, Cannon B, et al (2009) Chronic peroxisome proliferator-activated receptor gamma (PPARgamma) activation of epididymally derived white adipocyte cultures reveals a population of thermogenically competent, UCP1-containing adipocytes molecularly distinct from classic brown adipocytes. J Biol Chem 285: 7153–7164. 20028987 10.1074/jbc.M109.053942 PMC2844165

[21] Xue B, Rim JS, Hogan JC, Coulter AA, Koza RA, et al (2007) Genetic variability affects the development of brown adipocytes in white fat but not in interscapular brown fat. J Lipid Res 48: 41–51. 17041251 10.1194/jlr.M600287-JLR200

[22] Xue B, Coulter A, Rim JS, Koza RA, Kozak LP (2005) Transcriptional synergy and the regulation of Ucp1 during brown adipocyte induction in white fat depots. Mol Cell Biol 25: 8311–8322. 16135818 10.1128/MCB.25.18.8311-8322.2005 PMC1234324

[23] Coulter AA, Bearden CM, Liu X, Koza RA, Kozak LP (2003) Dietary fat interacts with QTLs controlling induction of Pgc-1 alpha and Ucp1 during conversion of white to brown fat. Physiol Genomics 14: 139–147. 12746468 10.1152/physiolgenomics.00057.2003

[24] Cederberg A, Gronning LM, Ahren B, Tasken K, Carlsson P, et al (2001) FOXC2 is a winged helix gene that counteracts obesity, hypertriglyceridemia, and diet-induced insulin resistance. Cell 106: 563–573. 11551504 10.1016/s0092-8674(01)00474-3

[25] Leonardsson G, Steel JH, Christian M, Pocock V, Milligan S, et al (2004) Nuclear receptor corepressor RIP140 regulates fat accumulation. Proc Natl Acad Sci U S A 101: 8437–8442. 15155905 10.1073/pnas.0401013101 PMC420412

[26] Seale P, Conroe HM, Estall J, Kajimura S, Frontini A, et al (2011) Prdm16 determines the thermogenic program of subcutaneous white adipose tissue in mice. J Clin Invest 121: 96–105. 21123942 10.1172/JCI44271 PMC3007155

[27] Hu HH, Gilsanz V (2011) Developments in the imaging of brown adipose tissue and its associations with muscle, puberty, and health in children. Front Endocrinol (Lausanne) 2: 33. 22649372 10.3389/fendo.2011.00033 PMC3355993

[28] Ohno H, Shinoda K, Spiegelman BM, Kajimura S (2012) PPARgamma agonists Induce a White-to-Brown Fat Conversion through Stabilization of PRDM16 Protein. Cell Metab 15: 395–404. 22405074 10.1016/j.cmet.2012.01.019 PMC3410936

[29] Rheinwald JG, Hahn WC, Ramsey MR, Wu JY, Guo Z, et al (2002) A two-stage, p16(INK4A)- and p53-dependent keratinocyte senescence mechanism that limits replicative potential independent of telomere status. Mol Cell Biol 22: 5157–5172. 12077343 10.1128/MCB.22.14.5157-5172.2002 PMC139780

[30] Li C, Wong WH (2001) Model-based analysis of oligonucleotide arrays: expression index computation and outlier detection. Proc Natl Acad Sci U S A 98: 31–36. 11134512 10.1073/pnas.011404098 PMC14539

[31] Saeed AI, Bhagabati NK, Braisted JC, Liang W, Sharov V, et al (2006) TM4 microarray software suite. Methods Enzymol 411: 134–193. 16939790 10.1016/S0076-6879(06)11009-5

[32] Kajimura S, Seale P, Tomaru T, Erdjument-Bromage H, Cooper MP, et al (2008) Regulation of the brown and white fat gene programs through a PRDM16/CtBP transcriptional complex. Genes Dev 22: 1397–1409. 18483224 10.1101/gad.1666108 PMC2377193

[33] Vernochet C, Peres SB, Davis KE, McDonald ME, Qiang L, et al (2009) C/EBPalpha and the corepressors CtBP1 and CtBP2 regulate repression of select visceral white adipose genes during induction of the brown phenotype in white adipocytes by peroxisome proliferator-activated receptor gamma agonists. Mol Cell Biol 29: 4714–4728. 19564408 10.1128/MCB.01899-08 PMC2725706

[34] Lee P, Greenfield JR, Ho KK, Fulham MJ (2010) A critical appraisal of the prevalence and metabolic significance of brown adipose tissue in adult humans. Am J Physiol Endocrinol Metab 299: E601–606. 20606075 10.1152/ajpendo.00298.2010

[35] Gilsanz V, Smith ML, Goodarzian F, Kim M, Wren TA, et al.. (2012) Changes in brown adipose tissue in boys and girls during childhood and puberty. J Pediatr 160: 604–609 e601. 10.1016/j.jpeds.2011.09.035 PMC3307823 22048045

[36] Wu J, Bostrom P, Sparks LM, Ye L, Choi JH, et al (2012) Beige adipocytes are a distinct type of thermogenic fat cell in mouse and human. Cell 150: 366–376. 22796012 10.1016/j.cell.2012.05.016 PMC3402601

[37] Shioda T, Fenner MH, Isselbacher KJ (1996) msg1, a novel melanocyte-specific gene, encodes a nuclear protein and is associated with pigmentation. Proc Natl Acad Sci U S A 93: 12298–12303. 8901575 10.1073/pnas.93.22.12298 PMC37985

[38] Prasad ML, Pellegata NS, Kloos RT, Barbacioru C, Huang Y, et al (2004) CITED1 protein expression suggests Papillary Thyroid Carcinoma in high throughput tissue microarray-based study. Thyroid 14: 169–175. 15072698 10.1089/105072504773297830

[39] Aldred MA, Huang Y, Liyanarachchi S, Pellegata NS, Gimm O, et al (2004) Papillary and follicular thyroid carcinomas show distinctly different microarray expression profiles and can be distinguished by a minimum of five genes. J Clin Oncol 22: 3531–3539. 15337802 10.1200/JCO.2004.08.127

[40] Yahata T, Shao W, Endoh H, Hur J, Coser KR, et al (2001) Selective coactivation of estrogen-dependent transcription by CITED1 CBP/p300-binding protein. Genes Dev 15: 2598–2612. 11581164 10.1101/gad.906301 PMC312794

[41] Svensson PA, Jernas M, Sjoholm K, Hoffmann JM, Nilsson BE, et al (2011) Gene expression in human brown adipose tissue. Int J Mol Med 27: 227–232. 21125211 10.3892/ijmm.2010.566

[42] Mochizuki N, Shimizu S, Nagasawa T, Tanaka H, Taniwaki M, et al (2000) A novel gene, MEL1, mapped to 1p36.3 is highly homologous to the MDS1/EVI1 gene and is transcriptionally activated in t(1;3)(p36;q21)-positive leukemia cells. Blood 96: 3209–3214. 11050005

[43] Nishikata I, Sasaki H, Iga M, Tateno Y, Imayoshi S, et al (2003) A novel EVI1 gene family, MEL1, lacking a PR domain (MEL1S) is expressed mainly in t(1;3)(p36;q21)-positive AML and blocks G-CSF-induced myeloid differentiation. Blood 102: 3323–3332. 12816872 10.1182/blood-2002-12-3944

[44] Seale P, Kajimura S, Yang W, Chin S, Rohas LM, et al (2007) Transcriptional control of brown fat determination by PRDM16. Cell Metab 6: 38–54. 17618855 10.1016/j.cmet.2007.06.001 PMC2564846

[45] Barbatelli G, Murano I, Madsen L, Hao Q, Jimenez M, et al (2010) The emergence of cold-induced brown adipocytes in mouse white fat depots is determined predominantly by white to brown adipocyte transdifferentiation. Am J Physiol Endocrinol Metab 298: E1244–1253. 20354155 10.1152/ajpendo.00600.2009

[46] Arch JR (2002) beta(3)-Adrenoceptor agonists: potential, pitfalls and progress. Eur J Pharmacol 440: 99–107. 12007528 10.1016/s0014-2999(02)01421-8

[47] Yadav H, Quijano C, Kamaraju AK, Gavrilova O, Malek R, et al (2011) Protection from obesity and diabetes by blockade of TGF-beta/Smad3 signaling. Cell Metab 14: 67–79. 21723505 10.1016/j.cmet.2011.04.013 PMC3169298

[48] Koncarevic A, Kajimura S, Cornwall-Brady M, Andreucci A, Pullen A, et al (2012) A novel therapeutic approach to treating obesity through modulation of TGFβ signaling. Endocrinology 153: 3133–3146. 22549226 10.1210/en.2012-1016 PMC3791434

[49] Fisher FM, Kleiner S, Douris N, Fox EC, Mepani RJ, et al (2012) FGF21 regulates PGC-1alpha and browning of white adipose tissues in adaptive thermogenesis. Genes Dev 26: 271–281. 22302939 10.1101/gad.177857.111 PMC3278894

[50] Bordicchia M, Liu D, Amri EZ, Ailhaud G, Dessi-Fulgheri P, et al (2012) Cardiac natriuretic peptides act via p38 MAPK to induce the brown fat thermogenic program in mouse and human adipocytes. J Clin Invest 122: 1022–1036. 22307324 10.1172/JCI59701 PMC3287224

[51] Vegiopoulos A, Muller-Decker K, Strzoda D, Schmitt I, Chichelnitskiy E, et al (2010) Cyclooxygenase-2 controls energy homeostasis in mice by de novo recruitment of brown adipocytes. Science 328: 1158–1161. 20448152 10.1126/science.1186034

[52] Madsen L, Pedersen LM, Lillefosse HH, Fjaere E, Bronstad I, et al (2010) UCP1 induction during recruitment of brown adipocytes in white adipose tissue is dependent on cyclooxygenase activity. PLoS One 5: e11391. 20613988 10.1371/journal.pone.0011391 PMC2894971

[53] Bostrom P, Wu J, Jedrychowski MP, Korde A, Ye L, et al (2012) A PGC1-alpha-dependent myokine that drives brown-fat-like development of white fat and thermogenesis. Nature 481: 463–468. 22237023 10.1038/nature10777 PMC3522098

