# Supplementary figures and images for: Human BAT Possesses Molecular Signatures That Resemble Beige/Brite Cells
Source: PLoS One. 2012 Nov 16;7(11):e49452. doi: 10.1371/journal.pone.0049452 (PMC3500293; doi:10.1371/journal.pone.0049452)

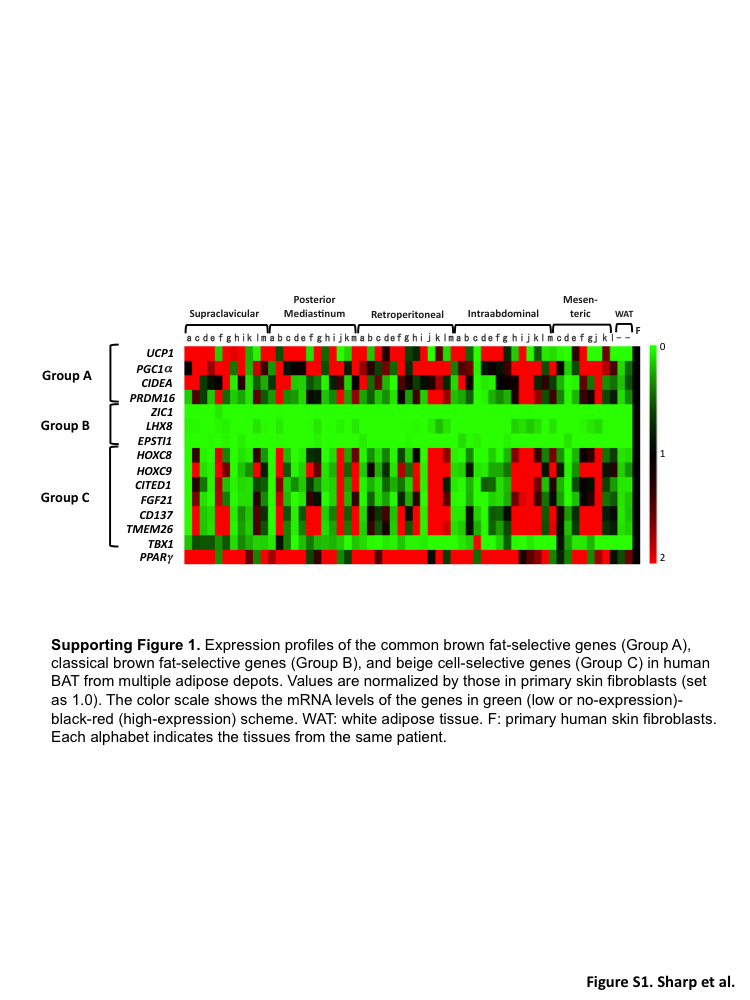

Supplement: Figure S1 — Expression profile after normalizing the each gene mRNA level with that of primary skin fibroblasts. (TIF) [file pone.0049452.s001.tif]
