# Supplementary material for: Human BAT Possesses Molecular Signatures That Resemble Beige/Brite Cells
Source: PLoS One. 2012 Nov 16;7(11):e49452. doi: 10.1371/journal.pone.0049452 (PMC3500293; doi:10.1371/journal.pone.0049452)
Supplement: Table S1 — Primer sequences used in quantitative real-time PCR. (PDF) [file pone.0049452.s002.pdf]

Supplementary Table 1
Primer Sequences used in real-time PCR

Gene	Species	Forward Primer	Reverse Primer
Car4	mouse	TACGTGGCCCCCTCTACTG	GCTGATTCTCCTTACAGGCTCC
Cidea	mouse	ATCACAACTGGCCTGGTTACG	TACTACCCGGTGTCCATTTCT
Cited1	mouse	AACCTTGGAGTGAAGGATCGC	GTAGGAGAGCCTATTGGAGATGT
Cox8b	mouse	GAACCATGAAGCCAACGACT	GCGAAGTTCACAGTGGTTCC
Lhx8	mouse	GAGCTCGGACCAGCTTCA	TTGTTGTCCTGAGCGAACTG
Fgf21	mouse	CTGCTGGGGGTCTACCAAG	CTGCGCCTACCACTGTTCC
Hoxa9	mouse	CCCCGACTTCAGTCCTTGC	GATGCACGTAGGGGTGGTG
Pgc1α	mouse	CCCTGCCATTGTTAAGACC	TGCTGCTGTTCCTGTTTTC
Prdm16	mouse	CAGCACGGTGAAGCCATTC	GCGTGCATCCGCTTGTG
Tbp	mouse	ACCCTTCACCAATGACTCCTATG	TGACTGCAGCAAATCGCTTGG
Ucp1	mouse	CACCTTCCCGCTGGACACT	CCCTAGGACACCTTTATACCTAATGG
Zic1	mouse	CTGTTGTGGGAGACACGATG	CCTCTTCTCAGGGCTCACAG
CD137	Human	AGCTGTTACAACATAGTAGCCAC	TCCTGCAATGATCTTGTCCTCT
CIDEA	Human	GGCAGGTTCACGTGTGGATA	GAAACACAGTGTTTGGCTCAAGA
CITED1	Human	CAACCTTGCGGTGAAAGATCG	GGAGAGCCTATTGGAGATCCC
CLDN1	Human	TGAAGTGCTTGGAAGACGATG	GGCAACTAAAATAGCCAGACCT
EPSTI1	Human	ACCCGCAATAGAGTGGTGAAC	GCTATCAAGGTGTATGCACTTGT
FGF21	Human	GCCTTGAAGCCGGGAGTTATT	GTGGAGCGATCCATACAGGG
HOXC8	Human	ACCGGCCTATTACGACTGC	TGCTGGTAGCCTGAGTTGGA
HOXC9	Human	GCCGGACTGTAGCGATTTTC	TATACCACGGACGACTGAGAG
LHX8	Human	GAATGACCTATGCTGGCATGT	AGTAGAATGGATGTGTCTCCCA
PGC1α	Human	CTGTGTCACCACCCAAATCCTTAT	TGTGTCGAGAAAAGGACCTTGA
PPARγ	Human	AGCCTCATGAAGAGCCTTCCA	TCCGGAAGAAACCCTTGCA
PRDM16	Human	CGAGGCCCCTGTCTACATTC	GCTCCCATCCGAAGTCTGTC
TBP	Human	CACGAACCACGGCACTGATT	TTTTCTTGCTGCCAGTCTGGAC
TMEM26	Human	ATGGAGGGACTGGTCTTCCTT	CTTCACCTCGGTCACTCGC
UCP1	Human	GTGTGCCCAACTGTGCAATG	CCAGGATCCAAGTCGCAAGA
ZIC1	Human	CACGCGGGACTTTCTGTTC	TGCCCGTTGACCACGTTAG
