# Supplementary material for: Human BAT Possesses Molecular Signatures That Resemble Beige/Brite Cells
Source: PLoS One. 2012 Nov 16;7(11):e49452. doi: 10.1371/journal.pone.0049452 (PMC3500293; doi:10.1371/journal.pone.0049452)
Supplement: Table S2 — Patient and sample information for the tissues used in the expression analyses. (PDF) [file pone.0049452.s003.pdf]

Supplementary Table 2

Tissue sample information

Patient Identification	Gender	Age	Tissues used in expression analysis
a	male	2 week	Supraclavicular<br>Posterior Mediastinum<br>Retroperitoneal<br>Intraabdominal<br>Thigh
b	male	3 year	Posterior Mediastinum<br>Retroperitoneal<br>Intraabdominal
c	male	11 week	Supraclavicular<br>Posterior Mediastinum<br>Retroperitoneal<br>Intraabdominal<br>Mesenteric
d	male	3 day	Supraclavicular<br>Posterior Mediastinum<br>Retroperitoneal<br>Intraabdominal<br>Mesenteric<br>Thigh
e	female	13 day	Supraclavicular<br>Posterior Mediastinum<br>Retroperitoneal<br>Intraabdominal<br>Mesenteric
f	male	6 week	Supraclavicular<br>Posterior Mediastinum<br>Retroperitoneal<br>Intraabdominal<br>Mesenteric<br>Thigh
g	male	6 day	Supraclavicular<br>Posterior Mediastinum<br>Retroperitoneal<br>Intraabdominal<br>Mesenteric<br>Thigh
h	female	5 day	Supraclavicular<br>Posterior Mediastinum<br>Retroperitoneal<br>Intraabdominal
i	female	2 week	Supraclavicular<br>Posterior Mediastinum<br>Retroperitoneal<br>Intraabdominal
j	female	14 year	Posterior Mediastinum<br>Retroperitoneal<br>Intraabdominal<br>Mesenteric
k	female	6 day	Supraclavicular<br>Posterior Mediastinum<br>Retroperitoneal<br>Intraabdominal<br>Mesenteric
l	male	18 year	Supraclavicular<br>Retroperitoneal<br>Intraabdominal<br>Mesenteric
m	female	3 month	Supraclavicular<br>Posterior Mediastinum<br>Retroperitoneal<br>Intraabdominal
