# Supplementary material for: Human BAT Possesses Molecular Signatures That Resemble Beige/Brite Cells
Source: PLoS One. 2012 Nov 16;7(11):e49452. doi: 10.1371/journal.pone.0049452 (PMC3500293; doi:10.1371/journal.pone.0049452)
Supplement: Table S3 — Genes enriched in rosiglitazone-inducible beige/brite cells. (PDF) [file pone.0049452.s004.pdf]

Supplementary Table 3
Genes enriched in rosiglitazone-inducible beige/brite cells

Gene Name	Accession	EntrezGene	fold change
carbonic anhydrase 4	NM_007607	12351	81.56
arylacetamide deacetylase (esterase)	NM_023383	67758	79.37
fibroblast growth factor 21	NM_020013	56636	26.9
acetyl-Coenzyme A acyltransferase 1B	BC019882	235674	26.19
RIKEN cDNA 2610528A11 gene	BF580962	70045	22.72
ADP-ribosyltransferase 4	NM_026639	109978	22.41
glycogen synthase 2	BC021322	633620	17.57
claudin 1	BB210412	12737	12.67
fatty acid binding protein 3, muscle and heart	NM_010174	14077	10.99
glycine-N-acyltransferase	BC010799	107146	10.95
carnitine palmitoyltransferase 1b, muscle	AF017174	12895	10.6
enoyl-Coenzyme A, hydratase	NM_023737	74147	8.78
cytochrome P450, family 2, subfamily c, polypeptide 70	BC025822	226105	8.54
claudin 23	BC019534	71908	8.44
tachykinin receptor 3	NM_021382	21338	8.25
Cbp/p300-interacting transactivator with Glu/Asp-rich carboxy-terminal domain 1	U65091	12705	7.71
T-cell receptor beta, variable 13	BF318536	269846	7.1
RIKEN cDNA 2810432L12 gene	BC013800	67063	6.95
disrupted meiotic cDNA 1 homolog	D58419	13404	6.4
Interleukin 17D	AI462269	239114	6.12
elongation of very long chain fatty acids (FEN1/Elo2, SUR4/Elo3, yeast)-like 4	BB829575	83603	5.72
solute carrier family 27 (fatty acid transporter), member 1	NM_011977	26457	5.2
keratin complex 2, basic, gene 1	NM_008473	16678	5.11
