# Supplementary material for: Human BAT Possesses Molecular Signatures That Resemble Beige/Brite Cells
Source: PLoS One. 2012 Nov 16;7(11):e49452. doi: 10.1371/journal.pone.0049452 (PMC3500293; doi:10.1371/journal.pone.0049452)
Supplement: Table S4 — Genes enriched in classical brown adipocytes (PDF) [file pone.0049452.s005.pdf]

Supplementary Table 4
Genes enriched in pre-exsting brown adipocytes

Gene Name	Accession	EntrezGene	fold change
epithelial stromal interaction 1 (breast)	AK017174	108670	182.5
expressed sequence AI447904	BM241008	236312	74.31
similar to SP140 nuclear body protein (predicted)	BC007193	620078	50.96
chemokine (C-X-C motif) ligand 11	NM_019494	56066	48.72
chemokine (C-X-C motif) ligand 5	NM_009141	20311	46.82
orosomucoid 3	NM_013623	18407	46.55
zinc finger protein of the cerebellum 1	BB361162	22771	45.24
interferon-induced protein with tetratricopeptide repeats 2	NM_008332	15958	41.99
myxovirus (influenza virus) resistance 1	M21039	17857	40.97
thymidylate kinase family LPS-inducible member	AK004595	22169	40.33
immunoresponsive gene 1	L38281	16365	38.27
thymidylate kinase family LPS-inducible member	AK004595	22169	37.24
interferon-induced protein 44	BB329808	99899	30.43
T-cell specific GTPase	NM_011579	21822	25.98
2 cells egg cDNA, RIKEN full-length enriched library, clone:B020014N01	C77655		25.53
interferon-induced protein with tetratricopeptide repeats 1	NM_008331	15957	24.29
interferon-induced protein with tetratricopeptide repeats 3	NM_010501	15959	22.76
radical S-adenosyl methionine domain containing 2	BB741897	58185	21.89
radical S-adenosyl methionine domain containing 2	BB741897	58185	20.98
lipocalin 2	X14607	16819	20.31
DNA segment, Chr 11, Lothar Hennighausen 2, expressed	AF316999	80861	20.21
intelectin a	NM_010584	16429	20.19
chemokine (C-X-C motif) ligand 10	NM_021274	15945	19.97
chemokine (C-X-C motif) ligand 1	NM_008176	14825	19.84
2'-5' oligoadenylate synthetase 2	AB067535	246728	19.61
2'-5' oligoadenylate synthetase-like 2	BQ033138	23962	19.52
epithelial stromal interaction 1 (breast)	BF020640	108670	18.45
methyl-CpG binding domain protein 1	AK007371	17190	18.36
interferon activated gene 203	NM_008328	15950	17.19
radical S-adenosyl methionine domain containing 2	BB132493	58185	16.96
myxovirus (influenza virus) resistance 2	BC007127	17858	15.78
cDNA sequence BC013672	BC013672	234311	14.4
breakpoint cluster region homolog	AI853148	110279	14.2
hypothetical protein LOC677168	AK019325	677168	13.9
preferentially expressed antigen in melanoma like 7	BG066968	347712	13.41
synuclein, gamma	NM_011430	20618	13.2
poly (ADP-ribose) polymerase family, member 14	BC021340	547253	12.82
ubiquitin specific peptidase 18	NM_011909	24110	12.61
transforming growth factor, beta receptor II	NM_009371	21813	12.51
chemokine (C-X-C motif) ligand 9	NM_008599	17329	12.39
signal transducer and activator of transcription 2	AF088862	20847	12.32
Kv channel-interacting protein 2	NM_030716	80906	12.29
guanylate nucleotide binding protein 2	NM_010260	14469	12.01
ubiquitin-activating enzyme E1-like	AK004894	74153	11.9
phospholipid scramblase 2	NM_008880	18828	11.88
RIKEN cDNA 9830147J24 gene	BC010229	229900	11.73
tripartite motif protein 34	AF220142	434218	11.7
serum amyloid A 1	NM_011314	20208	11.61
tubulin, alpha 8	NM_017379	53857	11.35
Forkhead box D1	BB662927	15229	11.29
macrophage expressed gene 1	L20315	17476	11.02
tripartite motif protein 34	AF220141	94094	10.96
tripartite motif protein 12	BM244351	76681	10.93
RIKEN cDNA 2310016F22 gene	BC020489	223672	10.77
tripartite motif protein 30	BG068242	20128	10.55
toll-like receptor 3	NM_126166	142980	10.44
RIKEN cDNA 9830147J24 gene	BM241271	229900	10.19
interferon gamma inducible protein 47	NM_008330	15953	10.11
Z-DNA binding protein 1	NM_021394	58203	9.92
mitochondrial ribosomal protein L21	AW824334	353242	9.77
adenosine deaminase, RNA-specific	AF291876	56417	9.75
interferon activated gene 203	BC008167	15950	9.7
hypothetical protein LOC677168	AK019325	677168	9.62
RAR-related orphan receptor gamma	AJ132394	19885	9.51
2'-5' oligoadenylate synthetase-like 1	AB067533	231655	9.3
guanylate nucleotide binding protein 2	BE197524	14469	9.26

serum amyloid A 1	NM_009117	20208	9.25
POU domain, class 4, transcription factor 3	NM_138945	18998	9.23
tripartite motif protein 30	AF220015	20128	9.18
histocompatibility 2, class II antigen A, alpha	BE688749	14960	9.17
tripartite motif protein 34	NM_030684	434218	9.16
interferon gamma induced GTPase	NM_018738	16145	9.06
RIKEN cDNA A430104N18 gene	AA254104	78591	8.97
toll-like receptor 2	NM_011905	24088	8.97
Purkinje cell protein 4	NM_008791	18546	8.95
PYD and CARD domain containing	BG084230	66824	8.9
toll-like receptor 3	NM_126166	142980	8.89
signal transducer and activator of transcription 2	AF088862	20847	8.87
Interferon activated gene 203	AA200306	15950	8.67
schlafen 8	BC024709	276950	8.57
clusterin	AV152288	12759	8.57
zinc finger protein of the cerebellum 4	NM_009576	22774	8.55
hyaluronic acid binding protein 2	AI035669	226243	8.53
clusterin	BB433678	12759	8.53
interferon inducible GTPase 2	NM_019440	54396	8.53
three prime repair exonuclease 1	NM_011637	22040	8.47
2'-5' oligoadenylate synthetase 3	AB067534	246727	8.43
clusterin	AV075715	12759	8.43
tripartite motif protein 30	BM240719	20128	8.25
cDNA sequence BC027127	BB085570	211739	8.19
RIKEN cDNA 1500011J06 gene	BC008229	208606	8.18
interferon activated gene 203	M74124	15950	8.14
ubiquitin-conjugating enzyme E2L 6	BC008238	56791	7.98
N-myc (and STAT) interactor	BC002019	64685	7.56
heat shock protein 1A	AW763765	193740	7.51
chemokine orphan receptor 1	BC015254	12778	7.47
promyelocytic leukemia	NM_008884	18854	7.43
serum amyloid A 2	NM_011314	20209	7.4
solute carrier family 9 (sodium/hydrogen exchanger), member 8	AF482993	77031	7.38
clusterin	NM_013492	12759	7.27
ankyrin repeat and SOCS box-containing protein 13	AF403041	142688	7.2
2'-5' oligoadenylate synthetase 1A	BC018470	246730	7.17
stathmin-like 4	NM_019675	56471	7.1
CD274 antigen	NM_021893	60533	7.04
torsin family 3, member A	NM_023141	30935	7.01
interleukin 18 binding protein	AF110803	16068	6.99
TRAF type zinc finger domain containing 1	AK003586	231712	6.95
interleukin 6	NM_031168	16193	6.95
ATP-binding cassette, sub-family A (ABC1), member 8b	AF213393	27404	6.87
nuclear antigen Sp100	U83636	20684	6.87
expressed sequence AI481105	BC025488	98999	6.85
chemokine (C-X-C motif) ligand 2	NM_009140	20310	6.83
torsin family 3, member A	NM_023141	30935	6.83
potassium voltage-gated channel, shaker-related subfamily, beta member 1	AK015412	16497	6.77
vomeronasal 1 receptor, A2	Y12724	22297	6.66
hypothetical protein 9530028C05	BQ175154	330256	6.66
guanylate nucleotide binding protein 4	NM_018734	55932	6.64
inter-alpha trypsin inhibitor, heavy chain 3	NM_008407	16426	6.62
immunity-related GTPase family, M	NM_008326	15944	6.55
receptor transporter protein 4	BC024872	67775	6.52
ceruloplasmin	BB332449	12870	6.51
a disintegrin-like and metallopeptidase (reprolysin type) with thrombospondir	BB658835	23794	6.47
expressed sequence AI451617	BG068242	209387	6.38
similar to interferon-inducible GTPase	BC020118	435565	6.37
TAF7-like RNA polymerase II, TATA box binding protein (TBP)-associated factor	AF285574	74469	6.33
DNA segment, Chr 11, Lothar Hennighausen 2, expressed	NM_030150	80861	6.2
RIKEN cDNA 0610011I04 gene	BC006049	66058	6.11
Synaptophysin	AV151500	20977	6.05
component of Sp100-rs	BB148221	114564	6.04
microrchidia 1	NM_010816	17450	6
ceruloplasmin	BB332449	12870	5.95
tumor necrosis factor receptor superfamily, member 11a	AK020374	21934	5.93
DNA segment, Chr 14, ERATO Doi 581, expressed	AK017107	52023	5.89
phosphorylase kinase gamma 1	NM_011079	18682	5.87
RIKEN cDNA 4833442J19 gene	AV002340	320204	5.83
serine (or cysteine) peptidase inhibitor, clade B, member 1b	AF426025	282663	5.81

zinc finger protein 455	AV172851	218311	5.8
ceruloplasmin	BB332449	12870	5.74
beta-1,3-glucuronyltransferase 1 (glucuronosyltransferase P)	BC023052	76898	5.73
interferon induced with helicase C domain 1	AY075132	71586	5.73
chaperone, ABC1 activity of bc1 complex like (S. pombe)	AK014605	67426	5.68
chemokine (C-C motif) ligand 5	NM_013653	20304	5.68
ret proto-oncogene	NM_009050	19713	5.67
coagulation factor VIII	NM_007977	14069	5.66
expressed sequence C79490	C79490	97296	5.63
chemokine (C-C motif) ligand 2	AF065933	20296	5.59
glutathione S-transferase, alpha 1 (Ya)	NM_008182	14857	5.57
schlafen 2	NM_011408	20556	5.55
hypothetical protein LOC620695	BB560177	620695	5.53
solute carrier family 16 (monocarboxylic acid transporters), member 2	AW105741	20502	5.53
killer cell lectin-like receptor subfamily C, member 2	NM_010653	16642	5.5
Kruppel-like factor 15	BC013486	66277	5.44
pregnancy-specific glycoprotein 28	AF113598	114871	5.4
poly (ADP-ribose) polymerase family, member 12	BM227980	243771	5.39
naked cuticle 2 homolog (Drosophila)	BC019952	72293	5.37
macrophage activation 2 like	BM241485	100702	5.37
histidine decarboxylase	BG072171	15186	5.36
chemokine (C-C motif) ligand 9	AF128196	20308	5.34
angiopoietin 2	NM_007426	11601	5.31
RIKEN cDNA 5830443L24 gene	NM_029509	626578	5.17
interferon regulatory factor 1	NM_008390	16362	5.17
annexin A8	NM_013473	11752	5.16
B-cell leukemia/lymphoma 3	NM_033601	12051	5.13
interleukin 1 family, member 9	AY071843	215257	5.11
chemokine (C-C motif) ligand 9	AF128196	20308	5.07
proteasome (prosome, macropain) subunit, beta type 10	NM_013640	19171	5.06
inter alpha-trypsin inhibitor, heavy chain 4	AK004893	16427	5.05
chemokine (C-C motif) ligand 7	AF128193	20306	5.01
